# Networked Learning in 2021: A Community Definition

**DOI:** 10.1007/s42438-021-00222-y

**Published:** 2021-03-25

**Authors:** Networked Learning Editorial Collective (NLEC), Lesley Gourlay, José Luis Rodríguez-Illera, Elena Barberà, Maha Bali, Daniela Gachago, Nicola Pallitt, Chris Jones, Siân Bayne, Stig Børsen Hansen, Stefan Hrastinski, Jimmy Jaldemark, Chryssa Themelis, Magda Pischetola, Lone Dirckinck-Holmfeld, Adam Matthews, Kalervo N. Gulson, Kyungmee Lee, Brett Bligh, Patricia Thibaut, Marjan Vermeulen, Femke Nijland, Emmy Vrieling-Teunter, Howard Scott, Klaus Thestrup, Tom Gislev, Marguerite Koole, Maria Cutajar, Sue Tickner, Ninette Rothmüller, Aras Bozkurt, Tim Fawns, Jen Ross, Karoline Schnaider, Lucila Carvalho, Jennifer K. Green, Mariana Hadžijusufović, Sarah Hayes, Laura Czerniewicz, Jeremy Knox

**Affiliations:** 1 Lancaster University, Lancaster, UK; 2 University College London Institute of Education, London, UK; 3 University of Barcelona, Barcelona, Spain; 4 Universitat Oberta de Catalunya, Barcelona, Spain; 5 American University in Cairo, Cairo, Egypt; 6 Cape Peninsula University of Technology, Cape Town, South Africa; 7 Rhodes University, Makhanda, South Africa; 8 Liverpool John Moores University, Liverpool, UK; 9 University of Edinburgh, Edinburgh, UK; 10 University of Southern Denmark, Kolding, Denmark; 11 KTH Royal Institute of Technology, Stockholm, Sweden; 12 Department of Education, Mid Sweden University, Sundvall, Sweden; 13 NTNU: Norwegian University of Science and Technology, Høgskoleringen 1, 7491, Trondheim, Norway; 14 IT University of Copenhagen, Copenhagen, Denmark; 15 Aalborg University Copenhagen, Copenhagen, Denmark; 16 University of Birmingham, Birmingham, UK; 17 Sydney School of Education and Social Work, Faculty of Arts and Social Sciences, The University of Sydney, Sydney, Australia; 18 Universidad Austral de Chile, Valdivia, Chile; 19 Open Universiteit, Heerlen, Netherlands; 20 University of Wolverhampton, Wolverhampton, UK; 21 Danish School of Education, Aarhus University, Aarhus, Denmark; 22 Centre for Educational Development, Aarhus University, Aarhus, Denmark; 23 University of Saskatchewan, Saskatoon, Canada; 24 University of Malta, Msida, Malta; 25 University of Auckland, Auckland, New Zealand; 26 University of Michigan, Ann Arbor, Michigan USA; 27 Warchée, Beirut, Lebanon; 28 Anadolu University, Eskişehir, Turkey; 29 Department of Education, Umeå University, Umeå, Sweden; 30 Massey University, Aotearoa, New Zealand; 31 University of Sarajevo, Sarajevo, Bosnia and Herzegovina; 32 University of Cape Town, Cape Town, South Africa

## Introduction (Networked Learning Editorial Collective)

Since the turn of this century, much of the world has undergone tectonic socio-technological change. Computers have left the isolated basements of research institutes and entered people’s homes. Network connectivity has advanced from slow and unreliable modems to high-speed broadband. Devices have evolved: from stationary desktop computers to ever-present, always-connected smartphones. These developments have been accompanied by new digital practices, and changing expectations, not least in education, where enthusiasm for digital technologies has been kindled by quite contrasting sets of values. For example, some critical pedagogues working in the traditions of Freire and Illich have understood computers as novel tools for political and social emancipation, while opportunistic managers in cash-strapped universities have seen new opportunities for saving money and/or growing revenues. Irrespective of their ideological leanings, many of the early attempts at marrying technology and education had some features in common: instrumentalist understandings of human relationships with technologies, with a strong emphasis on practice and ‘what works’.

It is now clear that, in many countries, managerialist approaches have provided the framing, while local constraints and exigencies have shaped operational details, in fields such as e-learning, Technology Enhanced Learning, and others waving the ‘Digital’ banner. Too many emancipatory educational movements have ignored technology, burying their heads in the sand, or have wished it away, subscribing to a new form of Luddism, even as they sense themselves moving to the margins. But this situation is not set in stone. Our postdigital reality results from a complex interplay between centres and margins. Furthermore, the concepts of centres and margins ‘have morphed into formations that we do not yet understand, and they have created (power) relationships which are still unsettled. The concepts … have not disappeared, but they have become somewhat marginal in their own right.’ (Jandrić and Hayes 2019) Social justice and emancipation are as important as ever, yet they require new theoretical reconfigurations and practices fit for our socio-technological moment.

In the 1990s, networked learning (NL) emerged as a critical response to dominant discourses of the day. NL went against the grain in two main ways. First, it embarked on developing nuanced understandings of relationships between humans and technologies; understandings which reach beyond instrumentalism and various forms of determinism. Second, NL embraced the emancipatory agenda of the critical pedagogy movement and has, in various ways, politically committed to social justice (Beaty et al. 2002; Networked Learning Editorial Collective 2020). Gathered around the biennial Networked Learning Conference,^1^ the Research in Networked Learning book series,^2^ and a series of related projects and activities, the NL community has left a significant trace in educational transformations over the last few decades.

Twenty years ago, founding members of the NL community offered a definition of NL which has strongly influenced the NL community’s theoretical perspectives and research approaches (Goodyear et al. 2004).^3^ Since then, however, the world has radically changed. With this in mind, the Networked Learning Editorial Collective (NLEC) recently published a paper entitled ‘Networked Learning: Inviting Redefinition’ (2020). In line with NL’s critical agenda, a core goal for the paper was to open up a broad discussion about the current meaning and understandings of NL and directions for its further development.

The current collectively authored paper presents the responses to the NLEC’s open call. With 40 contributors coming from six continents and working across many fields of education, the paper reflects the breadth and depth of current understandings of NL. The responses have been collated, classified into main themes, and lightly edited for clarity. One of the responders, Sarah Hayes, was asked to write a conclusion. The final draft paper has undergone double open review. The reviewers, Laura Czerniewicz and Jeremy Knox, are acknowledged as authors.

Our intention, in taking this approach, has been to further stimulate democratic discussion about NL and to prompt some much-needed community-building.

## Redefinitions

### Entanglement, Silence and Being in Education (Lesley Gourlay)

Thinking about writing this response, I was reminded of Latour’s famous analysis of what he described as the ‘four difficulties’ of Actor-Network Theory, ‘…the words ‘actor’, ‘network’ and ‘theory’ – without forgetting the hyphen’ (Latour 1999: 15). I cannot aspire to Latour’s critical acuity, but this term is composed of two words which regularly cause me considerable discomfort, for a range of reasons. However, the task is to consider them together in the context of the unfolding trajectory of NL, so I will focus on that challenge. Goodyear and colleagues provide a helpful critical review of the evolution of the term and associated work. They conclude with the following definition:

Networked learning involves processes of collaborative, co-operative and collective inquiry, knowledge-creation and knowledgeable action, underpinned by trusting relationships, motivated by a sense of shared challenge and enabled by convivial technologies. (Networked Learning Editorial Collective 2020)

The authors set out where they see the deficiencies of NL as it is currently configured, specifically that it fails to take account of emancipatory struggles and political imperatives in society more broadly.

My first point relates to my reservations about the term ‘networked’. As the authors acknowledge in their review, the question arises as to what these connections are actually *for*. I would argue that, via a laudable move away from a neoliberal ‘delivery’ mode of digital education, NL may have fallen into the same hole as higher education more generally—namely a collapse into pure process, a fetishization of interaction for its own sake, even a new version of what Biesta (2012) calls ‘learnification’. This, turbo-charged by an over-extended application of social constructivism—plus in my view the chill wind of unfounded educators’ guilt—can lead to what Macfarlane (2017) characterises as forms of student performativity, enactments of ‘engagement’ along narrow lines which fit a dominant set of Anglo-American discourses about ‘active’ student behaviour.

My second point is that it is precisely this fundamentally ideological preoccupation with process over content and situatedness which blocks progress in terms of linking to specific emancipatory struggles. At the risk of alienating my readership, I would contend that the overwhelming focus on ‘connections’ is not only profoundly humanist; it implicitly favours a particular type of human—confident, articulate, orientated towards observable ‘connections’—and implicitly unhindered by the frequent structural and symbolic violence suffered online by those of us considered less-than-human, such as women, people of colour, LGBTQ people, differently abled people and so on. The abstract and somewhat utopian nature of the definition may appear inclusive, but I would argue, unless problematised, only looks emancipatory from those already standing at the top of the triangle looking down.

In conclusion, I would argue that NL could benefit from a move away from process (and wish-fulfilment), towards a more ethnographic sensibility, opening up educational settings in terms of the actual, situated, more-than-human ‘mess’ of specific contexts, disciplinary content and cultures, and also the wide diversity of ways of engaging, some of which might value solitude, reticence, silence, and different ways of ‘being’ in education—digital or otherwise, connected or not.

### Another Look at NL (José Luis Rodríguez-Illera and Elena Barberà)

The joint position paper (Networked Learning Editorial Collective 2020) and this response are good examples of collaboration that the authors deem to be a distinctive feature of NL. They can also be considered as results—and certainly not the only ones—of biennial conferences that have developed and theorized on the concept of NL over the years. To an extent this collaboration is also a reflection of a crisis, of perceived necessity for change, and of the need to substantiate ideas about NL through some kind of a manifesto.

Any concept, theory, approach, or practice is set within a *field* and acquires much of its identity by contrasting itself with other competing theories and fields. NL is no exception to this dynamic. Jones (2015), whose work constitutes perhaps the most standard background reference for the manifesto, devotes his first chapter to distinguishing his approach from others (e-Learning and Technology Enhanced Learning in particular). In Table 1, NL intellectual foundations, Networked Learning Editorial Collective (2020) adopts a highly inclusive intellectual background of the field. It is possibly an overly inclusive one, since broadness arrives at the expense of specificity, creating greater theoretical dispersion and methodological difficulty. To a large extent, this broadness comes from the metaphor of the network through which learning is discussed. Nardi and O’Day (1999) defined ways of thinking about technology as a tool, text, system, and ecology. NLEC adopt a systemic-ecological approach and are interested in a comprehensive definition. However, these metaphors entail a ‘point of view’ contradiction between them that is difficult to resolve.

In any case, NL is not the first approach to have its own set of problems and contradictions while situating itself within other approaches. One may recall approaches beset by greater problems, as those based on behaviourist or cognitive rigid frameworks, such as Instructional Design or Educational Technology. Let us briefly look at some of the main problems with Networked Learning Editorial Collective’s (2020) definition of NL:

1. There is no reference to ontogenetic development, as if it does not exist. Perhaps the authors only contemplate adult learning. It is not that they consider children to be ‘small adults’, but given the changes affecting their education, children and adolescents certainly deserve some mention.
2. NL places much emphasis on collaborative learning; it is one of NL’s core principles, and one that we fully endorse. Nevertheless, among the many ICT-mediated dyads (learner-learner, learner-tutor, learning community-learning resources, and others), an important dyad is forgotten: the dyad which connects the learner to him or herself, to his or her mechanisms of acquisition, appropriation, and regulation of knowledge. Any learning which modifies forms of activity and cognitive schemes also requires acquisition. This acquisition—whether reflective or spontaneous, conscious or tacit—is mainly personal and ultimately modifies previous learning experiences marked by individual differences.

Questions raised and the avenues for development suggested by the Networked Learning Editorial Collective (2020) are very important and will encourage other authors to join NL, broaden the field, and add to the efforts reflected in their invitation paper.

**Table 1:** Design dimensions for NL experiences

Dimension	Description
Facilitation	To what extent were there facilitators working directly with learners?
Openness	To what extent was the learning experience open to any participants outside an institution, and were materials openly accessible?
Structure	To what extent was there structure that was planned and followed?
Voluntariness (related to structure)	To what extent was participation of learners’ voluntary versus part of something mandatory
Linearity (related to structure)	To what extent does the learning experience flow in a particular order?
Certification	Was there certification at the end for completion? How formal is this certification (e.g. accredited, assessed, informal?)
‘Eventiness’	To what extent are there clear deadlines and timed commitments?
Content vs process	To what extent is the learning experience designed around content/learning outcomes vs process goals? (Smith 2018)
Homogeneous learning path versus autonomous pathways	Is there just one pathway or multiple? (see Crosslin 2018)
Playfulness	To what extent were ‘fun’/elements of play used?
Collaboration	To what extent is collaboration built into the design of the learning experience?
Affective	To what extent is the affective dimension of NL encouraged, emphasised, recognised or centred?
Socially just economically	To what extent is the networked design emphasizing economic social justice principles, using tools and technologies accessible to a broad range of target learners with different infrastructure supports?
Socially just culturally	To what extent is the networked design emphasizing cultural social justice principles? Is there representation from diverse and especially marginalised cultures?
Socially just politically	To what extent is the networked design emphasizing political social justice principles? Are there diverse learners/teachers involved in the design of the learning experience? How much power do they have in decision-making ‘parity of participation’? (Fraser 2005)

### Redefining NL as a Multidimensional Spectrum? (Maha Bali, Daniela Gachago, Nicola Pallitt)

From our experience, design considerations, such as context, have become more complex and varied than during the early days of NL. Understanding the dynamics between these is important for designing NL experiences. Therefore, rather than a definition, we suggest a range of dimensions which characterise NL experiences, such as ‘open/closed, structured/unstructured, facilitated/unfacilitated, certified/uncertified, with/without date commitments, homogenous versus autonomous learning path, content vs process centric, serious vs playful and individual vs collaborative’ (Gachago et al. 2020). In this response, we add to them ‘affective’ (building on Cleveland-Innes 2012) because cognitive dimensions are often emphasised, but affective aspects are not always considered. As an *overarching* dimension, we also emphasize ‘socially just’ (building on Bali et al. 2020), because not all pedagogical decisions promote social justice on an economic, cultural, or political level (Fraser 1995) and many current NL definitions do not necessarily explicitly acknowledge social justice (see for example Networked Learning Editorial Collective 2020). See Table 1 for a list of design dimensions for NL experiences.

These dimensions are work-in-progress and are also intertwined. Also, importance of dimensions differs by context. Social justice considerations particularly are meta pre-design decisions and can/should be applied across other dimensions, e.g. when there is structure, whose interests does it serve? Are there affective or social justice implications around choosing a particular structure when designing for particular learning experiences? We invite others to add to this list as we continue to.

### A Redefinition Requiring a Political and Technological Focus (Chris Jones)

The definition of NL has been extremely robust and provided a framework for a productive and expansive body of work. Nevertheless it is timely to review the original definition and its origins and purposes. Furthermore the need for an article responding to the effects of the Covid-19 pandemic and its consequences for educational technologists/ies makes this redefinition extremely relevant and important. The emergency response to Covid-19 has highlighted the two issues I want to raise, firstly the kinds of technology that are used and how that impacts on educational practices and secondly the formal political framework within which NL takes place. My comments below should be taken in that context of a strong endorsement of the motivation behind a revision of the longstanding definition of NL.

The focus on technology disappears in the revised definition. The suggested definition only contains the terms ‘convivial technologies’ and ‘machines’ which stand in for these complex socio-technical issues. I would like to see how *technologies* (specifically digital technologies) shape and are shaped by human activity reflected in any revised definition.

The definition needs to emphasise the relationship to technologies, understood as socio-technical systems and to stress the role of *digital networks* as configurations that straddle both technical systems and human interactions—interactions between humans, between humans and machines, and in assemblages of both humans and machines. Digital technologies would be clearer than convivial technologies and more specific. It is important to say that suggesting NL *depends* on digital technologies is not to propose any binary oppositions (e.g. virtual–real). It is to acknowledge that the social forms of NL, and its focus on connectivity, rely on a range of affordances specific to digital technologies.

For this reason I propose this small but important change to the definition: replacing convivial technologies with *digital* technologies.

Just as NL depends upon technology, it also depends on politically shaped social and technological contexts. More directly, the digital technologies developed in the second half of the twentieth century, and their regulation, were conditioned by a political framework that both influenced, and was influenced by, new forms of deregulated political and economic systems. Libertarianism and radical forms of neo-liberal political economy were the formative influences on (and in part the outcome of) Silicon Valley technologies.

The revised definition argues that NL has roots in critical and emancipatory educational traditions which underscore a commitment to equity and social justice. It also has roots in the direct political engagement that led to institutional innovations such as The Open University. I think making the political implications of this more explicit helps answer another question raised in the redefinition—‘what the connections made in Networked Learning are *for*’. The article lists a range of issues that are currently neglected in NL including class, critical race studies, postcolonialism, indigenous knowledge, gender studies, queer theory, green and blue environmentalism, and sustainability. I argue that to address these issues requires an unambiguous engagement in formal politics because it will be through political decisions that the social and technological conditions within which NL functions will be set. It is only by way of formal political engagement that open discussion of these issues will be protected, and solutions can be found.

## The Curious Relationships Between Concepts and Agendas

### What Do Definitions Do? (Siân Bayne)

My response to the paper re-defining NL revolves around three questions. What is the value of definition? What are the effects of definition? And who gets to define?

The general thrust of the paper is to try to pin down a revised definition of what we mean when we talk about ‘NL’. This desire to define has been a long-running theme across NL conferences and publications, and the intention is clearly very good—a clear definition of a field galvanises scholarship, offers a point of reference to a community and supports a platform for change. Further, the direction which this re-definition takes—toward the political and social purpose of NL, its alignment with the concerns of social justice, its aspirations for a better way of talking about how we learn through and with technologies—is extremely welcome.

However, there is a sense here that in seeking to define and pin down the terms by which we describe the field, the authors fall into the trap of unintentionally working against these very aspirations. To define a field is necessarily to put boundaries around it, to determine which writings, conversations, people are ‘inside’ and which are ‘outside’. This is inevitable, and not a reason for choosing not to define. However it does mean that we need to be very careful about the terms of the definition, and I think the paper could do more to enact this care.

For example, the stated intellectual foundations of the field are not interrogated according to the justice-oriented terms of the re-definition. The list in Table 1, NL intellectual foundations (Networked Learning Editorial Collective 2020), is overwhelmingly male, white, Western and oriented to learning rather than social or critical theory (of course there are exceptions). NL has a long history, and it does need to be clear about the foundational scholarship that has shaped it. But if it is to re-define itself in more politically oriented terms, it also needs to interrogate its own basis in a certain kind of scholarship, situated in a particular set of injustices, inequalities and blind spots.

Another example is the relative anonymity of the author group—the ‘Networked Learning Editorial Collective’. Author collectives are not uncommon, but it’s quite rare for the names of authors of a piece to be hinted at but not made explicit. The paper acknowledges the input of a group of well-known and well-respected colleagues in the field, but it is unclear who is ultimately taking responsibility for the authorship of the paper, and therefore for the ownership of the definition. The unintended effect here is opacity rather than inclusion, leaving the reader to guess at the power dynamics at play in the authoring of the paper, and at where the line between the ‘insiders’ and the ‘outsiders’ sits.

Both these examples I think illustrate why we need to be so careful with field definitions—they create outsiders. In the first example, the existence of ‘outsiderness’ is left unacknowledged by the failure to critique the field’s own intellectual foundations. In the second example, outsiderness is left unacknowledged by the (however well-intentioned) obscuring of the responsibility of authorship except to ‘those in the know’.

Overall I am not convinced that we need to keep looping back to definitions of ‘NL’ in an attempt to ‘essentialise’ its terms. Do we really need the permission of a definition to pursue the concerns around learning, technology, social justice, climate crisis and colonisation that drive much current work in this area? The field has grown organically over the last 20 years, and its terms have shifted as new scholars and practitioners have come in with their own perspectives and interpretations of the broad term ‘NL’. Do we really need to draw new boundaries around this changed field? If we decide we do, let’s at least be explicit about its foundational terms and its exclusions, at a point when our geopolitical and socioeconomic futures need it more than ever.

### On Failing to Make Sense of a Field (Stig Børsen Hansen)

In Hansen (2018), I attempted to offer a definition of NL. Consulting authoritative expositions, the definition sought to respect a fundamental distinction between a stipulative and a descriptive definition (Gupta 2019). I unsurprisingly pointed to the scientific study of networks as one theoretically defining aspect of NL, and I drew on the works of Ivan Illich as a starting point for a narrative of the field. A fundamental assumption was that concepts are like boundary drawers (Wright 2010), and that a great part of their utility consists in allowing us to decide what falls on either side of the boundary.

While the collective reinforces the importance of the heritage from Illich, the definitional work in Hansen (2018) is summarized as one that sees NL as having little ‘intrinsic coherence’ (Networked Learning Editorial Collective 2020) and is seen to be neither constructive nor trying to improve matters. I shall attempt to point to where I most crucially seem to have taken a wrong turn. In doing so, I also suggest what it in this case might mean that a definition is ‘fit for purpose’ (Networked Learning Editorial Collective 2020). In Hansen (2018), I seem to have been misled by an emphasis on theory or thinker as a defining feature of a field. The guiding thought was Kuhn’s (1977) idea of an essential tension between seeking conceptual innovation in science and having a singular, sustained preoccupation with a theoretical concept or model. This is a tension in most scientific fields, and Kuhn originally underscored the importance of more singular and sustained modes of working, for the flourishing of the kinds of science he studied. As it is clear from Networked Learning Editorial Collective (2020), NL is much more of a bazaar, with a multitude of theoretical voices, than it is a cathedral.

If theory or thinker is unlikely to demarcate a field, then what is? One broad definitional theme emerges from the work of the Networked Learning Editorial Collective (2020): function. In short, functional definitions understand a thing in terms of what it does, and the collective sees a function for NL in wider society in virtue of addressing such topics as emancipation, justice and the possibility for scholars and practitioners to work ‘creatively’ and to ‘[build] resilience’ (Jones 2015: 241, in Networked Learning Editorial Collective 2020). Purposes can be subject to redefinition, and the collective wishes to emphasize ‘forms of emancipatory action research’ as well as advocacy in future work. The narrative, here in the shape of publications, is adjusted accordingly by singling out papers in the body of NL that align with this function. When stating that such approaches ‘[need] to find a place’, the definition of NL takes on an overtly stipulative character: NL is what we—a collective—think it should be. Moreover, the function also concerns what might be called the sociology of knowledge creation in higher education. In addition to its origin in the competitive environment of funding applications, NL as a field attracts third space professionals (Whitchurch 2008) and performs a role in arranging conferences and offering outlets for publications.

None of the proposed features of NL were ever academic *terra nullius*, and I doubt that they are when considered jointly. Attempts to demarcate NL via* negativa* continue (i.e., this is *not* blended learning and *not* online learning), but I suspect this academic field resists precise and effective boundary drawing beyond its institutionalization in academia combined with its subject. Even so, academics in NL study an increasingly widespread and in many ways important *practice* of networked entanglements, and continue to offer a theoretically and methodologically inclusive and edifying environment for sharing studies and insights.

### Redefining the Unredefinable? (Stefan Hrastinski)

The invitation paper is thought-provoking and covers lots of ground. As someone who has followed NL research from the outskirts and occasionally used the term in passing, it was especially interesting to read the discussion on what the connections in a network could be *for*. Although the term NL was defined decades ago (Goodyear et al. 2004), it is a term that has lived a life of its own, among practitioners and in other academic communities (Jackson and Temperley 2007; Lee et al. 2020). The theoretical understanding of the term NL might be constrained because it is so closely related to the everyday term networking.

According to the Cambridge dictionary (2021), networking has different meanings, such as ‘the process of meeting and talking to a lot of people, especially in order to get information that can help you’ *and* ‘the process of connecting two or more computers together so that they can share information’. These meanings have similarities with an early influential definition of NL: ‘learning in which information and communications technology (ICT) is used to promote connections: between one learner and other learners, between learners and tutors, between a learning community and its resources’ (Goodyear et al. 2004: 1). As evident in the commentary, the role of technology and formal education in NL is under debate.

Although simple, the early definition of NL is useful to encourage practitioners to move beyond content transmission and understand that networking is also a way to learn, and to think about how technology could provide opportunities for people to learn in networks across boundaries, such as time and space. Thus, I would argue that the core goal of the commentary is maybe not so much about redefinition, as it is ‘to open up discussion about the place of critical and emancipatory dispositions within current descriptions of networked learning’ (Networked Learning Editorial Collective 2020: 11). Trying to redefine a term that has been assigned with meanings is challenging, at least beyond a tight-knit academic community. I do not think that the commentary is so much about redefining a term that has already been assigned meanings among diverse groups of practitioners and academics, as it is about suggesting a research agenda that will hopefully influence the next decades of research on NL.

## Philosophical Foundations

### A Holistic and Non-dualistic Worldview as A Philosophical Foundation for a Definition of NL (Jimmy Jaldemark)

The need to redefine NL has been an ongoing discussion since the inception of the concept. In this discussion, the meaning of the idea of NL seems to be evolving and emerging. Recently, the Networked Learning Editorial Collective (2020) contributed to this discussion. Throughout the years, ontological and epistemological foundations of a particular worldview have saturated earlier contributions. This worldview builds on holistic and non-dualistic networked ideas of change, human agency and learning. A new definition of NL needs to continue building on ideas that align with such a worldview.

Dewey and Bentley (1949/1960) distinguish between interactional and transactional approaches to understanding human action. The interactional approach built upon a dualistic Newtonian worldview, where ‘action and reaction are equal and opposite’ (Dewey and Bentley 1949/1960: 68). Such approach focuses on a narrow study of human action that deemphasises cultural, historical, social, spatial, technological, or temporal conditions or motives. In short, such an approach comprises a dualistic and fragmentised understanding of change, human agency and learning by separating elements or variables from each other (Jaldemark 2010). A transactional approach differs from an interactional approach by embracing the messiness and networked complexity of change, human agency, and learning. The worldview in a transactional approach embraces the idea that ‘there are no separate elements … the whole is composed of inseparable aspects that simultaneously and conjointly define the whole’ (Altman and Rogoff 1991: 24). Therefore, cultural, historical, social, spatial, technological, and temporal aspects are dynamically involved in shaping networked human actions.

The worldview of earlier definitions of NL emphasises change, human agency and learning as complex holistic processes intertwined with and inseparable from the surrounding environment. A redefinition of NL should continue building on such a worldview and support transactional approaches. Therefore, it should avoid the inclusion of concepts linked to an interactional approach and a dualistic worldview. It needs to go beyond the boundaries of an interactional approach and deny dichotomies in the study of NL. In effect, it should include concepts that embrace the idea of NL as a boundless, hybrid and postdigital phenomenon that enables change, human agency and learning.

Applying such worldview suggests abandoning dualistic separations of the environment into several environments. Moreover, the fuzzy and unclear concept of interaction should be avoided and substituted with the application of more clear-cut concepts that differ between human-to-human interplay and humans’ interplay with resources in the surrounding environment. Finally, there is no such thing as offline or online human action. NL simultaneously embraces both offline and online aspects. Change, human agency and learning in a postdigital world are hybrid processes linked to the application of digital technologies.

To define, NL is a boundless, hybrid and postdigital phenomenon embracing the entanglement of cultural, historical, social, spatial, technological and temporal aspects of human actions and the world, and enabling change, human agency, and learning, through collaboration and dialogue between humans and through human interplay with aspects of the surrounding environment.

### NL Mirrored in Epistemologies (Logos for Episteme) (Chryssa Themelis)

In times of crisis such as the Covid-19 pandemic, people usually reflect to redefine their priorities and examine what is worth investing their time in. Networked learning was the title of my MSc at Lancaster University back in 2006 and the theoretical framework that dominated a life-long learning and research approach. Whenever a research question arose, my leading source of information was the networked connections, weak or strong ties with colleagues that I have been related to as part of my ‘onlife’. Whenever I was looking for partners for Erasmus calls in Higher Education, my social networks connected me to experts in the field that lead a similar online/offline path.

[A] future where the persistence of e-learning communities in higher education is not a fate one must choose for or against, but as a site for political, social, technological, pedagogical, and philosophical creativity directed toward ongoing understanding of dynamic, networked teaching and learning experiences. (Parchoma 2011: 81)

Starting from nothing, many ancient philosophers such as Plato, Socrates, and Aristotle dug deeper into the concept of episteme (knowledge) and ways to learn and reason (logics). In particular, Socrates was looking for the knowledge (*epistêmê*) in virtue of which the city is well-counseled: *demosophia*—the wisdom of the people (Parry 2020). Higher education institutions have the similar moral obligation to cope with the epistemologies (episteme and logics) to promote *epistemic fluency* of educators as well. Similarly, Markauskaite and Goodyear (2016: 20) have posited epistemic fluency as ‘a deep understanding of how knowledge works, the capacity to participate in the creation of actionable knowledge and a sense of how to reconfigure the world in order to see what matters more clearly and enable oneself, and others, to act more knowledgably’.

Another important aspect of episteme except epistemic fluency is to be aware of the epistemologies of ignorance. Epistemologies of ignorance is, rather, an ‘examination of the complex phenomena of ignorance’ (Sullivan and Tuana 2007: 1 in Bhatt and MacKenzie 2019), how fake news are constructed and disseminated for devious purposes against democracy (echo chambers, polarization and attention economy); how the digital wellbeing is threatened (depression, addiction, infringement of personal data) (Themelis and Sime 2020); and how ignorance, as a substantive epistemic practice in itself, is wilful and socially acceptable for a fragment of society to gain *epistemic advantage* (knowledge is power) (Alcoff 2007 in Bhatt and MacKenzie 2019).

Having the aforementioned concepts of episteme (epistemic fluency, epistemic advantage, and epistemology of ignorance) into consideration, NL is the episteme (knowledge seeking process) in which information, norms and behaviours are disseminated through epistemic relevant connections among social networks, resources and learners who have built epistemic fluency and mindful self-definition (awareness of role, content and impact) within transmedia ecologies.

### NL as Emergent Enacted Cognition (Magda Pischetola and Lone Dirckinck-Holmfeld)

In a recent collective effort of redefinition, NL has been associated with ‘processes of collaborative, co-operative and collective inquiry, knowledge-creation and knowledgeable action, underpinned by trusting relationships, motivated by a sense of shared challenge and enabled by convivial technologies’ (Networked Learning Editorial Collective 2020). In this theoretical contribution, we present the result of a dialogue between an old-timer and a newcomer to the field, which brings about a critical reflection about the abovementioned definition.

First, the ‘networked’ concept presents some shortcomings. If the theoretical bases of the NL movement are—among others—sociomaterial studies (Barad 2007; Fenwick 2015), the network should not be used as a metaphor, but rather in an ontological perspective, which focuses on sociotechnical/sociomaterial entanglements, and connects knowing with being (Dall’Alba 2009). Thus, we ask: does the network *always* generate collaborative, co-operative and collective processes of knowledge creation? Critical analysis of the last decade have recognised the bitter overcoming of democratic utopias (Buckingham 2020; Morozov 2011), as we see increasing exploitation of collective data (Selwyn 2010) by tech-monopolies that need to constantly reinvent their business, through the network (Jandrić and Hayes 2020; Williamson et al. 2020). In this paper, we suggest exploring the network ontologically, as a living and dynamic ecosystem (Pischetola and Miranda 2019), which is supported/created by constant exchange of information among its parts. This means considering each new information as ‘the difference that makes the difference’ in the network (Bateson 1972). The core notion of *emergence* can explain the complex process of knowledge-creation (Davis and Sumara 2008; Miranda and Pischetola 2020): ‘networked’ can be understood as ‘emergent’.

This brings us to the second critical aspect of the NL redefinition, which concerns the very meaning of ‘learning’. In fact, in an ecological/complex/sociotechnical perspective, when participants of a living ecosystem engage meaningfully in the process of knowledge-creation, this engagement generates *change* or, said otherwise, learning (Bateson 1972). This process takes place in a unique situation and through the coupling of brain, body, and environment (Merleau-Ponty 1962). In this approach, known as enactivism (Varela et al. 1991), ‘learning’ can be framed as ‘situated and embodied cognition’. This aspect is present in the concept of NL since the original formulation in 1998.

However, the aspects of enaction related to learning deserve more attention. Technologies, for example, seem to have been naturalised as platforms that enhance the process of learning or ‘convivial tools’ for social growth (Networked Learning Editorial Collective 2020). Do technologies *enhance* learning, *mediate* learning or do they *interfere radically* in learning? If we aim at considering technologies not merely as neutral tools (Feenberg 2003; Heinsfeld and Pischetola 2019), but as *agentic matter* (Haraway 1991) embedded with values (Selwyn et al. 2019), we need not only to acknowledge their active role in learning but explore how interactions with technologies (Kopcha et al. 2020) entail a different quality of value, material texture, information, aesthetics, conviviality, and environment to which we couple our bodies and brains in a relational designed NL practice. In other words, we ask: how is learning taking place *in* the network and *with* the network?

## Social Justice and Emancipation

### Social Justice in a Network of Sociotechnical Networks (Adam Matthews)

The provocation to rethink NL for a post-pandemic world lists social justice as an area for further incorporation into the diverse and well-established field. The pandemic itself has brought the concept of the network to the fore as a network entanglement which is biological, social, cultural, digital, and networked (Honigsbaum 2020; Matthews 2020a; Price 2020). Pre-pandemic social injustices (i.e. Waller et al. 2018; Reay et al. 2005; Savage 2015) have been heightened by the virus and subsequent social and economic lockdowns (Hu 2020; Murat and Bonacini 2020; Templeton et al. 2020). But what is the part of the technical in the sociotechnical network? Dismissing technology as neutral and ‘tool-like’ misses out a complex assemblage of human and non-human actors and the structures and agencies which technologies afford. It is clear that technology is not neutral where existing inequalities are reproduced by historical data and such technologies ‘act’ in machine learning, software and algorithms (Eubanks 2017; Gray and Suri 2019; Noble 2018).

Social and technical networks underpin the Network Society (Castells 2000; Pescosolido 2007; van Dijk 2020). Incorporating these networks and not thinking about them independently provides important perspectives on sociotechnical assemblages of the postdigital university (Gourlay 2015; Gourlay and Oliver 2018). A closer relationship between the social and the technical is provided by An and Oliver’s (2020) model of relational thinking across humans-education, human-technology, and education-technology. Moreover, Beckman et al. (2018) have developed a networked approach to technology, education, and social justice using Bourdieu’s network-like field, habitus and capital.

The application of Bourdieu’s theory of practice offers educational technology research, a tool to recognise the differing technology experiences that contribute to digital inequality, while highlighting the problematic nature of policy and curriculum that view technology as a socially, culturally and politically neutral vehicle for the simple acquisition of meritocratic outcomes. (Beckman et al. 2018: 201)

Students, teachers, designers, developers, policy makers and technology bring their habitus (cultural, economic and social capital) to many fields of sociotechnical network assemblages. Within these networks, thinking of digital technologies as mere tools to be used (Matthews 2020b), automatically enhancing learning (Bayne 2015), and students as simply users (Ramiel 2019), is problematised as substantive, essentialist and at the extreme technologically deterministic.

A network of sociotechnical networks also sees new policy actors. EdTech experts and policy makers produce ‘fast policy’ (Williamson 2019) impacting upon the sociotechnical network assemblage. This network of sociotechnical networks is growing further, in university settings, the degree and those carrying out teaching is being unbundled (McCowan 2017; Morris et al. 2020) into specialist roles of expertise with their own habitus and fields incorporating commercial interest and pedagogic views. This further broadens the network across new actors and organisations.

Re-emphasising the socio(logy) in the sociotechnical network takes us to the basis of the discipline—structure and agency. Who has agency in a complex sociotechnical network of actors? Theories of social constructivism, technological determinism, actor-network, postdigital, and postphenomenology (Matthews 2021) trace such agencies. Identifying agency from design and development through to use (Carvalho et al. 2019) provides a research trajectory to trace structure and agency in complex networks in new and interdisciplinary ways (i.e. Network Science, see Barabási and Pósfai 2016). The design and engagement with a network of networks then, is not so human and user centred but interrelated between human and non-human mediation (Aagaard 2017) requiring values of equality and justice in such designs (Forlano 2017).

### Topology, Posthumanism, Technology (Kalervo N. Gulson)

I am a neophyte to NL. I am an education policy scholar, with an interest in Science and Technology Studies. In the below comments, I am responding primarily to the final line of the paper, about ‘open questions about organizational and policy issues, which need deeper exploration’ (Networked Learning Editorial Collective 2020). These comments may provide possible ways of beginning that exploration.

My first thought is that for a field that talks about networks, NL needs more thought about what theories of power would be needed in any redefinition. For example, it strikes me that aiming to do emancipatory action research requires a theory of power that is congruent with networks. I wonder if it would be useful to examine the work in human geography that has emerged on power and topology, or what Allen (2011: 284) calls ‘power-topologies’ that are ‘not so much positioned in space or extended across it, as they compose the spaces of which they are a part’. This approach aims to highlight not only the relationships between bodies and things, but also what makes up these relationships. Organisationally, it is a theory of power that seems congruent with NL—it would allow for the positions of people (e.g. learners, educators) and things (e.g. institutions, technologies) to be understood as co-creating spaces of learning. In the policy area, this work that, can be loosely characterised as network governance studies, has looked at new education policy networks, including how ideas move and the importance of place, and the new actors involved in governing, including technology companies (Gulson and Witzenberger 2020; Lewis and Hardy 2017). Perhaps, the notion of power topologies would provide a conceptual tool to examine organizations and policies that is congruent with the field of NL.

My second thought is that organizational and policy issues are also issues of agency, and as such also to do with not only where we locate that agency (as in the above point about networks), but also who or what is agentic. Some preliminary points that follow this are that we can think about non-human ‘learners’ as parts of networks (AI fields such as deep learning is one such area), and therefore, it might be useful to think about posthumanism and related theories of technology. Obviously, the field of Actor Network Theory, and related areas are important here, as are concepts of technology that challenge our ideas that it is separate from the human, and rather see technology as imbricated with social, cultural, and political life (Haraway 1991; Mackenzie 2002) What does it mean for the idea of agency in NL if there are forms of (semi)automated systems like some AI? It could mean accepting that technology is not deterministic, but also that technology is uncontrollable and even accepted forms of control, such as regulation, may not be able to limit automated systems (Roden 2015).

### Towards a Manifesto of Struggle for Everyday Networked Learning (Kyungmee Lee and Brett Bligh)

This article is a welcome attempt to correct weaknesses in previous definitions of NL, reflecting societal and technological changes gaining prominence in recent times. We applaud the continuing commitment to criticality that has been a hallmark of the field. For decades, the NL community consciously distinguished itself from neighbouring research fields. Central was an attempt to *position* our understanding of educational relationships mediated by technology: against an explicitly societal backdrop of wider issues.

NL, in acknowledging and engaging with actual societies, does not shy away from issues of politics, inequality, and injustice. It is no accident that the emancipatory claims of NL are often framed using heavy names like Freire, Foucault, or Marx, albeit often taking-for-granted aspects of their conceptual heritage (e.g. Lee 2018). Our actual societies, of course, are fast developing: economically, technologically, culturally. Recent cultural developments (such as Black Lives Matter) have starkly emphasised structural injustice, and societal discourses about technology have highlighted how networked relationships can perpetuate or even reinforce such injustice (cf. Nagle 2017).

Critical engagement with wider societal issues is missing from the new conceptualisation in the invitation (Networked Learning Editorial Collective 2020). To suggest, at this moment, that NL is underpinned by ‘trusting’ relationships and enabled by ‘convivial’ technologies is naïve. This type of normative understanding neither adequately acknowledges the *challenges* of developing trust among people from different social, cultural, and political backgrounds; nor how skewed are technologies and their impacts on different people. It occludes that networks, whether digital or otherwise, do not only enable but disenable, producing many agonies for humans in actual society.

We argue that any NL definition needs to encompass lived experiences and the dynamics of struggle in daily practice. ‘Ordinary’ educators and researchers—including ourselves—face many challenges and dilemmas when working across disparate settings and with diverse students: dilemmas that may obstruct our attempts to foster NL or even be exacerbated by those attempts. As Ellsworth (1989) suggested years ago, attempts by practitioners to apply abstract-utopian principles rarely *feel* empowering. Thus, we contend, for the new definition be useful, it needs to better reflect the realities of ‘everyday NL’, and to foster a sense of *shared challenge*, rather than abstract ideals.

How, then, might we circumscribe ‘everyday NL’? The invitation to redefinition laudably invokes the idea of *manifesto*. Perhaps we might rediscover the ‘minimum–maximum’ structure of nineteenth century critical manifestos (e.g. Marx and Guesde 1880)? In such documents, the ‘minimum’ section demarcates basic demands: if *these* criteria are not met, then we might refuse to categorise a given phenomenon as NL *at all*. In standing opposed *en bloc* we would collectively orient ourselves towards wider societal debates. The ‘maximum’ section, by contrast, states ultimate future ideals: *those* criteria we strive towards, while emphasising the difficulty of their attainment. ‘Everyday NL’ might be understood as that conflicted practice which occupies the zone-in-between those minimum and maximum definitions. The field might work to highlight and explore the shared challenges and (often difficult) practice dynamics of those working in that zone.

One challenge for NL researchers is how to project normative visions while *differentiating* themselves from dominant discourses in educational regimes, which often seek to co-opt and neuter ostensibly radical demands. Previous definitions, which welcomed novelty and (Foucauldian) abnormality (cf. Lee 2020), to some extent achieved that goal. We believe that any new definition should *definitively* emphasise that critical-practical posture which has so far distinguished us from those myriad other groups projecting ideas of ‘future learning’. By mapping and navigating *shared challenges* within a clear zone of investigation, we might be able to do so.

### Towards the Inclusion of Global, Local and Sustainable Views (Patricia Thibaut)

In the first decade of the twenty-first century, technologies and particularly social network sites started to change the landscape of social, informational, political and economic practices. Educational practices were not an exception, as technologies were also introduced in classroom spaces. There have been, however, contrasting views about the impact and prospect of the use of technologies for learning. Within the education research community some signalled the potential for new ways of learning, more horizontal, collaborative and democratic, but others saw technologies as another tool to add to the teaching and learning repertoire, or in some cases, to replace teachers. After two decades of research and the global pandemic, the hype of positive views has been counterbalanced by the negative effects related to the use of technology, such as the datafication of education, digital divide, and increased awareness of how technology affects humans.

However, it is clear now that technologies have changed the way we live and work. Year 2020 showed that without technology, people at workplaces, universities, schools and other learning spaces would not have been able to continue to connect, work and learn. Interestingly, platforms for conference-calls such as Zoom or Meet, which have been around for years, were uncommon in educational institutions and workplaces. Thus, the global pandemic shifted the research landscape—from typically small, isolated case studies to a global sample of synchronized activity, within similar topics. Researchers in places as scattered as Chile and Australia, are asking similar questions. How to support teachers and students in their teaching and learning processes in remote emergency education and NL?

These present times are calling us to finetune the definition of NL. An early definition emphasized connections between individuals, learning materials, and learning community (Goodyear et al. 1998). More recently, aspects of space, activity, epistemic and social structures, agency, and purpose were highlighted (Goodyear and Carvalho 2014). As Yeoman (2016: 40) stresses, ‘where the digital and physical merge—in learning—and activity is strongly anchored in a particular place yet travels out of, into and through this permeable space in ways that are only possible via networked technologies’. The focus on networks offers an important contribution to help the understanding of co-operative, collaborative and community aspects in learning. The design lens helps to integrate aspects of learning that often are investigated in isolation, such as the social, epistemic, and set design, and how these elements relate to the emergent activity of learners.

Considering the evolution of the term ‘NL’ and the sudden transition to emergency remote teaching in 2020 (Hodges et al. 2020), we can now speak of a real global movement. It continues to be important to address ethical issues, and issues of identity, agency, and privacy in education. What is more, most research on the use of technologies for learning still tends to privilege certain areas of the world (Thibaut and Carvalho 2020). The current moment offers a valuable opportunity to turn our attention to the global south and bring a more diverse voice to the conversation. The challenge is, however, to understand a global phenomenon without losing sight of the particularities of culture and location. And avoid falling into stereotypes that are commonly attributed to what is not familiar. Finally, another critical question is, How do we move from an anthropocentric towards an ecosystem view of learning, in which a definition of learning—and its associated consequences—also include purpose and the need to adapt to more sustainable ways of living?

## Who/What Gets In? Who/What Is Out?

### Networked Learning, a Diversity Perspective (Marjan Vermeulen, Femke Nijland and Emmy Vrieling-Teunter)

NL is usually defined as the natural emergence of learning ties between people, based on their learning needs (cf. Nijland et al. 2018). Through means of interaction and shared activity, these learning ties facilitate and enable a change in cognition and behaviour. We perceive networked learning as a multi-level phenomenon, always including both the individual and the collective level (Vermeulen 2016). The interplay between these levels defines learning outcomes: collective or individual processes lead to collective and individual outcomes, and these processes and outcomes are thoroughly intertwined with the community that is constructed through and constituted by these learning ties.

Grounded on this interplay perspective, NL is inherently stemmed from diversity. Diversity sparks a process of sense-making in which learners attempt to align their individual or collective identity with those of others. This process can be seen as a mechanism of breakdown and common ground (Rajagopal et al. 2017). Breakdown can be described as a conflict of perspectives forcing the individual to reflect on ongoing activity. The search for common ground that follows is a sense-making process used to remedy the breakdown, which initiates an amended individual and collective perspective (Castelijns et al. 2004).

However, our research shows that this sense-making process is affected by the nature and degree of diversity that is experienced. In our studies (Nijland et al. 2018; Vermeulen and Nijland 2021; Vrieling-Teunter et al. 2019) into structured NL, in which both educational professionals and novices collectively participate in knowledge construction, diversity appears to be both the spark and the snuffer of this sense-making process. When aims for participation are collectively experienced as too diverse, for example when students collaborate with educational professionals in collective knowledge construction, but at the same time must complete an individual assignment, breakdown occurs but is not always remedied in sense-making, hindering alignment in a collective perspective (Vermeulen and Nijland 2021; Vrieling-Teunter et al. 2019). In other cases, great diversity in individual knowledge, experience and organisational background does result in breakdown but is followed by an ongoing sense-making process in which collective alignment is sought but never found. This dysfunction causes participants to leave resulting in the breakup of learning ties (Vermeulen and Nijland 2021).

Diversity appears to be a crucial factor in NL, but its effect can be described as parabolic. Too little diversity prevents breakdown and obstructs sense-making processes, while too much diversity results in non-remedied breakdown which may ultimately lead to the breaking up of learning ties. Both ends of the diversity spectrum snuff a collective sense-making process. However, we believe that too much diversity can be mitigated, for instance by fostering feelings of connectedness and equality during the collective search for common ground. Research into the effects of diversity should focus on exploring factors that counteract the negative effects and enhance the positive effects of great diversity.

### Social Media Fatigue and the Dilemma of Divergence (Howard Scott)

To be redefined NL must ask who is not there and seek to understand and integrate those who are excluded. In seeking to read the convergence and engagement activity and contributions of those in the network, NL fails to capture the penumbral and liminal thinking that is in the minds of those at the outer edges—the outliers, lurkers, and peripheral participants (Lave and Wenger 1991). NL theorists must confront the notion of *divergence*, which is to say those off the network map or those who have literally fallen through the net. They have been called peripheral, but this is a deficit as if they are lacking the nous of digital literacies or are victims of the digital divide. In reality, divergence is a choice and ambivalence can be a profound turning away and rejection of groupthink or consensus—what is called *Social Media Fatigue* (Scott 2018): properties of digital dissonance, which reside with those who do not see any value in community residence, with digital hegemony, by playing the game or joining the network. They are likely to seek their own communities elsewhere, which is clearly problematic for educators working with social learning models or who endorse any situated practices that are collaborative and co-operative.

These peripheral outliers constitute natural challenges to the thinking of a status quo—and there is no doubt that any NL community forms its own hierarchies. For instance, Holmes and Meyerhoff (1999) suggested that participants may be peripheral, but once they subscribe to the codes of a community they always and naturally gravitate towards the core. A status quo that claims its voice and views as representative of consensus, because what seems to be necessary for NL is to embrace and incorporate a plurality or views and voices. Therein, the divergent who reject and turn away are unlike a concentric circle or sub-domain, but another territory altogether outside the network—one that is fragmented, rather than clustered and disruptive, rather than cohesive. In some ways, these observations reflect the nihilistic spirit of much anti-social media in the contemporary era, where culture wars, disinformation and trolling become common practice. These comments should lead us to consider the ‘insider/outsider’ domains and question how and why those terrains diverge.

### Becoming Part of a Network (Klaus Thestrup and Tom Gislev)

#### Being in a Process

In the discussion about a redefinition of NL, we suggest to focus on how a network becomes a network. Based upon several projects involving schools and pre-schools inside and outside Denmark and Europe, where the participants to a large extent had not been part of networks before, one could talk about a process where the single school is not in any formalized networks and might not have any experience or consciousness about the potential of NL (Thestrup et al. 2018). Then they start to reach out to platforms and people locally, regionally, and globally to make the first contact. This might lead to the establishing of what we call a *flexible meeting place* (Gislev et al. 2020), where the participants reflectively experiment with how and where to communicate using media at hand.

#### Using Body and Space

The communication between the participants in the network can be combinations of intertwined analogue and digital processes conducted in synchronous and asynchronous ways. This includes many different technologies, spaces, and actions. NL does not only take place in front of a screen on a laptop, but also while dancing, playing, and experimenting using bodies situated in local contexts or using materials, tools, processes, and traditions in a workshop. All this obviously takes place in different tempi and different ways around the globe, yet it is all the time happening in a process, where more and more people are increasingly connected. The local and the global become interconnected as well, and NL might take place in both formal and informal arenas inside and outside schools and universities. It is not given in advance how technologies should be used, by whom and for what purpose, but it should be open to testing and dialogues in the emerging network.

#### Understanding Networks as a Media Ecology

We suggest that establishing contact to a network, i.e. connecting to other nodes in a network, is a process of entangled physical and digital probes, approaches or advances, that are situated in an emerging common space, more often than not mediated by technology. Technology, not being neutral, but multistable (Ihde 1990), mediates the perceptions and actions of the participants (Verbeek 2005), and by that co-shapes the space, the connections, and the network. We also suggest that such a learning network is an aggregation of multiple tools in a changing media ecology, and this points towards that learning through connections in networks. Being part of a NL community requires not just skills and competences regarding communication and social interaction, but also a profound understanding of the technology and skills and competences regarding designing and redesigning the network.

We therefore suggest that the partners involved in NL can be understood as *experimenting communities*, where the purpose is to experiment with and reflect upon the processes of becoming a community involved in NL. Communication and production can take place while unfolding life and dealing with local and global challenges and fascinations.

### Recognizing the Value of Mediating Experts in NL (Marguerite Koole)

Conceptualizing a definition that captures the nature of learning across the complex socio-material entanglements respectful of current, diverse contexts and purposes is an arduous task. Since the 1990s, NL scholars have endeavoured to balance issues of social justice, situatedness, critical reflexivity, responsibility, collaboration, and human-material relationships. As noted in ‘Networked Learning: Inviting Redefinition’ (Networked Learning Editorial Collective 2020), there are some areas within the NL literature that are undertheorized. One such area is the role of teachers.

A group of authors from the University of Edinburgh recently published a short book called *The Manifesto for Teaching Online* (Bayne et al. 2020). In it, they critique the *learnification* of education in which the learner is considered an independent, self-motivated individual who is able to manage and ‘curate’ (Selwyn 2016: 65) their own learning. In the process, the teacher becomes a mere facilitator and, taken to extremes, is deprofessionalized. Education ‘reduces the project of education entirely to the notion of learning and the learner’ (Bayne et al. 2020: 87). Within my own context here in Canada, many educators continue uncritically to promote the notion of *learner-centeredness*; few consider how such language supports neoliberal agendas using so-called *neutral* digital technologies to cut labour costs and systematically scale up enrolments. ‘High-quality education... is inherently complex, subtle, and various, making the subjection of teaching to the procedural fantasies of standardization and routinization framed as best practice highly problematic.’ (Bayne et al. 2020: 28) As Selwyn (2016: 73) argues, the role of ‘mediating experts’ remains crucial. Yet, by its very name, networked *learning* draws focus to the learner.

Within a socio-materialist perspective, subjects within a learning assemblage can take on multiple roles. For example, in studies using the community of inquiry model (Garrison et al. 2006), learners have been observed to enact teacher presence. I would add that non-human entities within a learning assemblage can take on multiple roles as *both/either* learner *and/or* teacher. (AI is an obvious example.) A socio-materialist approach could extend our understanding of the co-shaping, meshwork of relationships within learning assemblages.

Furthermore, NL might also strive to achieve a blend of/or sensitivity towards both individualist (humanist, progressivist) and collectivist performativities. Rather than NL promoting ‘*connections: between*’ (Networked Learning Editorial Collective 2020), a definition of NL could propose to examine ‘*interrelationship amongst* people, sites of learning and action, ideas, resources and solutions, and time, space, and media’ thereby avoiding suggestions of binaries and move towards multiplicity. Acknowledgement of multiplicity may enhance efforts towards social justice as it expands our awareness and acceptance of how agents within assemblages can flow between ontological categories.

### Criticality and Criticism of Trustful Relationships (Maria Cutajar)

The proposed redefinition of NL is an attempt to extend the existing succinct definition which for over 20 years served us well notwithstanding the criticism. This redefinition and the criticism epitomize deepening discernment and healthy advancement of the field. I also understand the need for an elaborate redefinition at a time when at large the term is picking up as a fashionable buzzword and at risk of becoming stripped of its core meaning in being pulled into the folds of political, economic, and techno-salvation discourses.

This redefinition highlights cooperative, collaborative and collective inquiry efforts for knowledge construction, development and value creation. Distinctions between the online and offline, the physical and virtual, the synchronous and asynchronous, the formal and non-formal, are messy and difficult to set apart in our conviviality with technology, with human others, and with ourselves, as we shift and drift across time, spaces, media, and realities. There is highlighted the centrality of collective effort for learning (and teaching) and the call for trustful relationships for upholding this. Crucially, relations for learning need to be understood as presided by critical reflexivity hence creating e-quality (Beaty et al. 2002) along with a valuing of democratic processes, inclusion and diversity (Ryberg et al. 2012).

The authors claim that challenging issues relating to humanistic and post-humanistic aspects of networked learning in the past were generally overlooked except for some sporadic works such as that of McConnell (2006). With respect to post-humanistic aspects, this can be safely claimed. It is only in recent times and the increasingly visible and deepened interfacing and interactivity of technology and the organic (including humans) that posthumanism has come to the fore. On the humanistic perspective, I note that for many years I have been going back to studies that specifically focus on the dark side of networked learning. Hodgson and Reynolds (2005) drew attention to the challenges in trying to build the aspired trustful interhuman relationships. Trehan and Reynolds (2002) exposed problems of intolerance for diversity and exclusion. Ferreday and Hodgson (2010) put a spotlight on the oppression and suppression that may arise leading to ‘tyranny of participation’ (Ferreday and Hodgson 2008).

It was this strand of NL research and my observations researching practice that led me to see NL as an aspiration to perfection (Cutajar 2014) even if a worthy one to pursue. Developing NL calls for a critical stance paying attention to the many shapes and forms of digital divides (Grant and Eynon 2017) and constructive responsiveness to both social presences and social absences (Öztok 2019). The darker side of NL experience needs to be discerned along with the more positive perspective, understanding both as a spectrum of variation in space, place, and time.

Coming at a time when the world is struggling to find sustainable ways out of environmental and climate change problems alongside a crippling Covid-19 pandemic crisis which forced teaching and learning to the virtual spaces or nothing at all, this redefinition (and this collective exercise) is deemed a significant just-in-time endeavour. It may well act as a beacon. It is a redefinition expanding what NL stands for. Hopefully, it proves to be a powerful means taking forward NL practice, research and theory development for many years to come no less than its predecessor, which in its simplicity and humble beginnings brought us thus far.

### The Way Forwards—Collaboration, Coercion or Exclusion? (Sue Tickner)

I qualify as an ‘old-timer’ as mentioned in the call for responses. I was a student of Peter Goodyear’s on the first iteration of the online distance MSc Information Technology and Learning in 1989. I was also a contributor to the Manifesto for E-learning released at the Networked Learning Conference (Beaty et al. 2002) which stressed the potential of technology to open barriers, widen access and support democracy. I am very conscious of the need to keep digital equity and social justice at the forefront of the definition.

Czerniewicz (2018), pre-Covid-19, drew attention to the fact that the global marketplace for online higher education was increasing some aspects of inequality. The ‘pivot’ to emergency online teaching (Hodges et al. 2020) was a necessary response to the pandemic, but one which carries great danger of exacerbating that slide into inequality. With many overseas students still unable to return to University, there is a financial imperative to retain numbers, more positively expressed as ‘maintaining a commitment to our students’. However, without adequate attention to the design of the learning experience these remote students are easily marginalised.

I support the idea of a Manifesto as a call to attention, to stop ‘going further in the same way’. I have often said that my first experience of NL changed my life. This is no exaggeration, both for my career-path and my views about the goals of education. NL is transformational, requires commitment with the ‘whole self’, and therefore vulnerability, trust, and the belief that one’s voice will be listened to. It entails ‘a commitment to *collaborative inquiry and joint action in the face of shared challenges*’ (Networked Learning Editorial Collective 2020) (emphasis from the original).

This is not easily achieved for a learner who is ‘zoomed-in’ to a predominantly face-to-face class, even with dedicated attention to their needs. I have experienced feelings of disempowerment and exclusion myself when joining meetings remotely whilst others are ‘co-present’. A lack of attention to the screen on the part of the physically present colleagues results in increasingly desperate and ‘out of synch’ attempts to put questions to the chat, until one gives up on trying to be a fully engaged participant. The disconnection is even more acute where there are cultural differences and access issues involved, as with foreign students joining domestic students on campus in the same course. If we believe that learning is socio-cultural, and situated, then building shared culture and collaboration in learning environments is crucial.

Dedicated classrooms equipped with large screens, workstations for learners in mixed-mode groups and sufficient skilled facilitators can make this model work, but it requires significant additional attention—a laptop at the back of the class is not NL.

It is not enough to simply ‘connect’ remote learners to an established community. If we are to offer NL opportunities to global communities, (rather than delivering online content), we need an intentional focus on universal design, cultural inclusion, and digital equity.

### Thumbs Up: Sophisticated Cats Under-cover (Ninette Rothmüller)

‘Thumbs up’, her voice cracks through the instable connection. ‘Show me your thumbs up if you have your work board out. We will do equations’. Rustling, her teacher’s voice breaks up and my daughter hums, audio off, humming as if bees cruised right through the room that has no name. On Saturday, my daughter, seven years old, looked puzzled. Taking turns, looking at me, looking at her play area—finally, taking a deep breath, ‘Mama, on Saturdays, is this room my classroom or is it my play area?’ This is not funny; deep crease between my daughter’s eyebrows, lost in too many spaces, locked into our apartment.

The cat, so her teacher had ruled, cannot be in her room during class hours. Wait, what? Who just decided where our cat can sleep? And who is going to explain that to the cat now? Go for it, there’s those members of society that don’t give a … about remote teaching etiquettes. Our cat is one of them. And where is that ‘other room’ where the cat is supposed to be? A classroom, a play area, a space for the cat? And, seriously, just how many quiet working spaces with zipping fast Internet connections for screen-tied children and adults can a tiny studio apartment have?

I turn and look at the weekly school report that just dropped into my junk mail. Ah, let’s see what has my seven-year-old daughter learned this week. At home she taught me how to juggle and I taught her how to crochet. Ah, yes, I should not have included this here, because what’s that going to do? At school, in front of her screen, sitting in the cat-free room that has no name, she learned how to ‘drag and drop’. Now who would have thought that the word ‘drag’ would take the back door and sneak into our children’s school report? I chuckle, looking at the hump under my daughter’s blanket. From Mondays to Fridays that blanket is located in her classroom. If you look very closely the hump goes up and down and up and down. It’s breathing… It’s our cat going to school ‘under cover’, big bright Cheshire cat grin on his mouth. Go, Alice, go, go straight to Wonderland through the screen you must pass, take your cat and your juggling balls, don’t stop humming.

Equations? Ah, I forget about them for a while. Or wait. Equations, let’s do some, shall we? The Latin word ‘equ’ means nothing else but ‘equal’. This can easily be understood when looking at the word equation, because both sides on an equation are ‘equal’ to one another. What if the screen is an equation? What if both sides of it were equal to one another? Just kidding—we cannot even get to the point where those located on one side of the screen are equal to one another. Forget it then. Wait, I was supposed to propose a word. My daughter is still humming. Audio off, lips shut, her teacher has no idea that she hums her way through equations. Revolution. Let’s call it a revolution. Révolution means to ‘turn around’. Let’s turn it around, let’s turn it up-side down and inside out. Education that is.

## Navigating Networks

### Online Networks as Ecologies of Learning (Aras Bozkurt)

Being social requires developing connections in a community, which means that networks are essential structures, insofar as they are the means by which humans build, nurture, and sustain their connections. Humans are social and so are the networks they create or engage in. Broadly speaking, networks are emergent, complex, chaotic, dynamic, self-maintained and self-organized structures, consisting of nodes and connections among these nodes. From a system perspective, a network itself can be a node and be connected to different networks. In this sense, networks are multidimensional and multilayered, and as learning is situated and context-dependent, the way one connects and sees the networks defines their roles in the learning process.

In learning networks, knowledge is distributed and decentralized. As learners produce and consume knowledge, they create networks that are living systems, that is, ecologies of learning. In these spaces, the individual or collective interactions we engage in while experiencing learning can involve living and non-living entities, a process that requires embracing post-humanist views. Considering that we are social beings and that these networks are shaped through our decisions, these networks are beyond simple digital binary structures, but rather, dynamic living organic spaces. In these living spaces, learners can form digital identities, build online communities, and grow their social capital.

As argued by Connectivism (Siemens 2005) and Rhizomatic Learning (Cormier 2008), learning is a non-linear process and it is self-determined (Blaschke et al. 2021). As there are no predefined paths, learners are given agency, which provides them the opportunity to take responsibility for their own learning and to build their own personal learning environments (PLEs) in a less-structured, more independent, autonomous space. From this perspective, it can be argued that NL is emergent, and that learning is defined by the needs of learners. Learners can navigate through the networks to better meet their learning needs by plugging in and out of different networks or nodes, cross-pollinating among multiple dimensions, paths, and layers of networked learning ecologies. The strength and promise of online networks lie in the abundance of connections, which liberates knowledge and democratizes learning. Overall, based on the abovementioned characteristics, NL can be seen as an extension of critical pedagogy, liberating education by empowering learners, promoting learner agency, and ensuring autonomy in the quest for meaningful learning experiences.

### Exploring the Metaphor of the Network (Tim Fawns and Jen Ross)

In ‘Networked Learning: inviting redefinition’ (Networked Learning Editorial Collective 2020), NL is presented as a community that studies the entanglements of ‘students, teachers, ideas, tasks, activities, tools, artefacts, places and spaces’, with increasing attention to ‘critical and emancipatory educational traditions … equity and social justice and … more sustainable forms of living’. While digital technology is an important consideration, it is increasingly not a discriminating factor given that this engagement is central to many current approaches. Instead, we respond to the ways in which ‘collaborative engagement in valued activity’ comes about, and ask: How do we define networked learning so that the network metaphor does not close down other kinds of topology?

Drawing from social topological theory, a network is a space made up of elements and the ‘well defined relations between them’ (Mol and Law 1994: 649). Networks are created by transporting ideas, creating old similarities in new places and homogenising and stabilising relations and practices, even as the elements within the network remain heterogeneous. Yet, it is not clear that stability and similarity are always what we should aim for, in working for a more socially just and equitable future for learning. For example, more peripheral nodes can only be functionally part of a network if certain conditions are met. In the case of education, this might be sufficient infrastructure, academic literacy in relation to dominant conventions, shared values, and local support networks in addition to the broader but more remote network.

Other spatial metaphors may offer more flexibility. Fluid space, for example, consists of ‘transformation without discontinuity’ (Mol and Law 1994: 658), such that identities cannot be neatly determined, nor ‘inside’ and ‘outside’ easily distinguished (660). In such a configuration, learning does not collapse in the absence of the network or any given learning space (Lamb and Ross 2021), because learning always spills out of its formal structures (Fawns 2019) and is not dependent on any particular node, mode of communication, or interface. To understand this, we need to look at local conditions and the fluid practices into which precisely defined nodes dissolve when they cannot conform.

Returning to the redefinition of NL, we ask, how do we support fluidity while maintaining the valuable coherence and momentum of the NL community? For us, the answer lies in the theoretical flexibility of collaboration and connection. While the network metaphor seems to emphasise identifying and understanding the nodes across which learning is distributed, the relations between them, and the wider context in which they are located, we can also consider more fluid conceptions of learning that are not contingent on the perpetuation of the network. By allowing non-conformity and fluidity of identity, structure, and relations, perhaps nodes and networks can learn from, without conforming to or replicating, the shapes and practices of others.

### What Are the Connections for? A Redefinition of NL from a Multimodal Layer Perspective (Karoline Schnaider)

More knowledge is needed in NL research on the connections between the tools and other resources used and the places and activities involved in that use. Besides, further explorations are necessary around the connections between designable things and learning, and how teachers and students react to designed things in meaning-making practices (Goodyear et al. 2014). However, recent interests in the NL field have identified a need to understand ‘what the connections are for’ (Networked Learning Editorial Collective 2020), which is missing in the existing definitions. Thus, a redefinition of NL is required that bridges the conceptual gap that exists today by focusing on the intimate ties between human meaning-making and technologies in a detailed and comprehensive way. For example, how technologies are shaped and re-shaped by human activity recognizes the multifunctional and multirepresentational formations of the different technologies and the complex practices in which they are configured and used by teachers and students in educational environments. A multimodal layer perspective has been fruitful for equally recognizing the different layers of technologies and human activities in the complex semiotic systems of learning settings (Schnaider et al. 2020).

The multimodal layers (MLs) relate to the dialectical and non-dualistic associations between technologies and their properties (the digital visual user interfaces, DVUIs) and human meaning-making (the cognitive processes) through five components. The *technologies* are used in combinations of hardware and software (Ravelli and van Leeuwen 2018). Technologies have *functional* (physical and symbolic sign-systems that orient actions through primary, secondary, and tertiary mediating levels) (Wartofsky 1979) and *semiotic properties* (sign-systems used in sign-making activities) (Jewitt 2017). The meaning-making is related to levels of mediation in actions (the Wartofskyan taxonomy) and sign-making (Bezemer and Kress 2016), which result in *modes of representation* in various *activities* between humans as well as between humans and the settings. In other words, the ML links the mediators to mediation through the concept of sign-systems (Schnaider et al. 2020).

An ML perspective focusing on technologies and humans through sign-systems is linked to the notions of human representations and the relations between form and meaning (Kress 2010; Wartofsky 1979). Representations signify a circular and intimate relationship between the external world, manifested with meaning by humans and simultaneously perceived and interpreted by them, and the internal mental processes, which produce and establish new representations to be used between people and between people and the given input (Vygotsky 1978). From a technology angle, DVUIs are designed and inhere physical and symbolic sign-systems that are in constant flux with different configurations of hardware and software (Ravelli and van Leeuwen 2018) and can orient actions and sign-making processes alternately. The properties get expanded from their meaning potentials and affordances depending on humans’ interpretation and needs, who are the agents in developing technology-enhanced practices and technology design (Kaptelinin and Nardi 2009). From a human perspective, people produce sign-systems when they perceive and interpret what is prompted to them by DVUIs and other humans in activities and are the signs of the meaning made visible in modes of representation.

An ML approach addresses ‘what the connections are for’ and renews the NL ideas from a multimodal standpoint to include things-to-things/things-to-human/human-to-human relations within a detailed and comprehensive framework (Goodyear et al. 2014; Schnaider et al. 2020). More knowledge of the nature of the technologies and human meaning-making through technology use can guide both design thinking and learning design and model future technology use and implementation.

### Learning to Navigate Networks of People and Things (Lucila Carvalho)

NL provides a language and a way to conceptualize learning activity as deeply grounded on *connections* between people, ideas, and things. The new definition of NL has been carefully crafted to emphasize processes of collaboration and participation, foregrounding knowledge creation and action (Networked Learning Editorial Collective 2020). It also nicely emphasizes the importance of trusting relationships and conviviality, whilst still maintaining at its core, the significance of the word *connections*. The use of the term *connections* seems still crucial here, inviting a nuanced view of learning. It makes us ponder on how we often rely on one another, and/or on things, for living and learning (Hodder 2012), on how we might gain access to information, or on how processes of participation, co-creation, and knowledge building are facilitated (Hodgson and McConnell 2019). Overall, it brings the importance of *learning to connect* to the fore. In order to develop more cohesive and sustainable societies, we all need to connect to others, to work together, so that we can successfully tackle the wicked problems of our lifetime—the climate crisis, people’s displacement, poverty, and Covid-19. We need to understand that what happens at one side of the world, often affects and reverberates elsewhere, through a chain of interdependent links. We need to see ourselves as part of a global network.

I have been particularly interested in the NL roots on Freire’s critical pedagogy (1972). This highlights the potential of connections for human growth, agency, and empowerment, whilst suggesting that the notion of NL goes beyond what can be achieved through the collective when people work together in shared enterprises of knowledge creation, towards also supporting and enhancing people’s individual experiences and personal growth, which are deeply enriched through these collective encounters and shared exchanges.

Importantly, NL not only allows us to articulate connections between people and things, or to notice their influence on individual and collective encounters. But it calls for a particular perspective on teaching and learning, centred around ways of facilitating the development of people’s ability to figure out what are the best tools and social strategies that may support engagement in successful collaborations, in ways that might sustain their participation within the networked structures of contemporary societies. Careful design for NL involves understanding assemblages of tools, people, and tasks, but also how these in turn may facilitate emergent learning activity (Goodyear and Carvalho 2014). Design for NL involves finding ways of supporting educators and learners figure out how to best navigate the intricate networks of our modern times.

### Designing for NL Within a Bachelor of Nursing (Jennifer K. Green)

I am an experienced teacher and leader in professional development and higher education settings with an interest in the use of technology for teaching and learning for over 10 years, yet I see myself as a relative newcomer to NL. The Covid-19 pandemic required immediate transition to fully online delivery, providing an opportunity to reflect deeply on connections between technology and learning. The way that my pivot team and I addressed this transition is discussed in Green et al. (2020). Our challenge for 2021 is to transform an emergency response into a cohesive and planned learning experience that optimises student learning, and aligns with, and could be assessed against, the NL principles outlined by Networked Learning Editorial Collective (2020).

NL principles related to collaboration, participation and knowledge-creation are underpinning our current development plan to offer a contiguous, hybrid learning environment to approximately 145 undergraduate students in three geographically separated year two Bachelor of Nursing cohorts. At present, due to containment of Covid-19 at the Aotearoa New Zealand border, we are able to offer face-to-face sessions. However, given the experience of continuing and restrictive lockdowns currently facing much of the world, we are proposing a new design, which involves simultaneous live streaming alongside face-to-face sessions thereby facilitating a smooth transition to fully online should this be required.

Course re-design includes group arrangements and allocation of time for collaborative, co-operative and collective inquiry supported by a variety of learning resources. This includes initial individual and group readiness quizzes to develop and assess baseline understanding. Then our learners apply their emerging understanding into case studies, whilst using a variety of convivial technologies, managed within our learning management system (e.g. PollEverywhere, Google JamBoard, Padlet, and Google Docs), discussions and resources from Patient/Service User informational platforms. NL principles are helping us set a scene to encourage learners to co-create person-centred, culturally appropriate, nursing care plans informed by Te Whare Tapa Whā (Durie 1985),^4^ a holistic, indigenous model of wellness.

Do our learners perceive these opportunities as beneficial? Our hope is that by moving beyond offering a lecture presentation after which learners might say, ‘I know this and that’, our approach might result in learners saying, ‘I understand the significance of this and can apply knowledge to person-centred care in a cohesive way’. As we delve into designing for NL (Goodyear and Carvalho 2014), we are searching for ways to create an environment in which each learner perceives that their learning needs are being addressed without waiting for subsequent hindsight to confirm this. How might we overcome the prevailing view of some learners that solely lecture-based is best? How might we convey the value of participation and co-creation as a community? How can we audit the effectiveness of our approach in our NL environment?

### Leveraging the Interconnectedness of the Twenty-First Century (Mariana Hadžijusufović)

Based on Ivan Illich’s (1971) proposal of ‘learning webs’ as a model for people to network their own learning, the concept of NL has flexibly expanded periodically requiring new definitions that would encompass the new realities, needs, expectations, and current states of affairs. NL has been characterized by an interest in the virtual and digital aspects of networked technologies, often focusing to online courses with individuals sitting at home and connected to other learners via their computers. As today’s education has become increasingly postdigital (Jandrić et al. 2018), the notion of network serves as an important framework for understanding learning in our era (Knox 2019).

Connectivism has been presented as a ‘learning theory for the twenty-first century’, closely linked with technological changes such as pervasiveness of various networked technologies and other mechanisms for aggregating and filtering information (Ryberg et al. 2012). Nowadays, NL is widely understood as a form of online learning that employs technology to connect individuals and/or groups and enables the transfer of information and knowledge between educators and learners. Asking Ryberg et al.’s (2012) question whether NL is about connectivity or collaboration, the answer is both. NL has a great deal to offer to those who want a distinctive label for such an ambitious conception of education (Networked Learning Editorial Collective 2020).

It is a pity that it took a global pandemic to reinforce the importance of NL for a modern society which strives for knowledge. The ongoing pandemic can be considered a catalyst that emphasises the need for educational change towards more flexible models and practices that best respond to the complexity and unpredictability of the interconnected yet still fragile society (Rapanta et al. 2020). Amongst others, it introduces new biodigital challenges (Peters, Jandrić, and McLaren 2020) which intersect with NL’s focus to social justice and equality. It is therefore time to better theorise the connections between developments in technology, inequality, and education, while also striving to actively design technologies that facilitate more equitable futures for all (Selwyn 2020), alongside with a commitment to collaborative action in the face of shared challenges.

## Conclusion (Sarah Hayes)

Upon being invited to write a conclusion to this critical, collective, dialogue, I read each response with an interest that goes well beyond the 500-word limit permitted for each submission. I immediately felt interconnected (networked, if you will) with each author and keen to know more about what brought each contributor into the NL community in the first place. For me, it was via my MSc in Advanced Learning Technology at Lancaster University, which I undertook on top of my work in a UK university, more than 15 years ago. For each module, I would leave my two young children and travel 200 miles to attend taught residentials, to eagerly learn from tutors who were founding members of the NL community. Back then I was also a ‘neophyte to NL’ (Gulson this article). I would receive journal articles via post to supplement online materials, in a broader context that I would now recognise as postdigital (Jandrić et al. 2018). These were like treasures because they supported my critical resistance to a managerialist policy rhetoric surrounding phrases like e-learning and Technology Enhanced Learning, that persisted in isolating technology from the rich interconnected human labours of teaching and learning (Hayes 2019; Matthews 2020b). The NL community has persevered instead with more emancipatory understandings of relationships between humans, technologies, and learning. These reach far beyond instrumentalism, in that they are ‘boundless, hybrid and postdigital’ (Jaldemark, this paper). As such, NL is indeed analogous to ‘a bazaar, with a multitude of theoretical voices’ (Hansen, this paper) all contributing to a ‘knowledge seeking process’ (Themelis, this paper).

Now though, in our messy postdigital world that is seeking routes out of a global pandemic, it is vital to debate the direction for these theoretical voices from NL, but not to draw boundaries that would colonise the community from ‘concerns around learning, technology, social justice and climate crisis’ (Bayne, this paper). Indeed, it would seem that the organic growth of NL that Bayne refers to, is a particular strength of NL that too tight a focus on terminology could confine. Whilst NL could have been constrained by its relations with the ‘everyday term networking’ (Hrastinski, this paper), I was always interested too in the idea that ‘networked learning can be considered the outcome of convergence’ (Jones and Steeples 2002: 3). It seemed to me that ‘convergence’ could be inclusive of all of the factors surrounding each of us that influence learning. This was once described as a ‘coming together of distance and place-based learning in a new form’ (Mason and Kaye 1990). This is a form that is forever changing though, as it perpetually converges with a ‘postdigital positionality’ (Hayes 2021) experienced by each of us, wherever we may be located.

Many authors here agree that it is vital ‘to encompass lived experiences and the dynamics of struggle in daily practice’ (Lee and Bligh, this paper). It is through engaging with, and responding to, concrete, fast-developing societies (Lee and Bligh, this paper) in research and in design of learning that NL can be distinguished from more deterministic agendas. This includes also a focus towards ‘the negative effects related to the use of technology, such as the datafication of education, digital divide, and increased awareness of how technology affects humans’ (Thibaut, this paper). There are opportunities also to encompass many ‘cultural developments (such as Black Lives Matter) that have starkly emphasised structural injustice, and societal discourses about technology’ and ‘have highlighted how networked relationships can perpetuate or even reinforce injustice’ (Lee and Bligh, this paper; Hayes 2021).

Therefore, at the core of this paper is the meaning we take from words themselves, as these come into play in discourse about technology and related matters. Like technologies, words are never neutral. Often words that describe learning technology agendas are strongly underpinned by a rationality in educational policy discourse that reflects the current neoliberal structuring of our political economy (Hayes 2019; Matthews, 2020b). This structuring has caused important debates that cut across culture and digital technologies to influence learning, to remain disconnected and isolated from each other, across many decades.

It would seem though that we could yet be entering a new and exciting period where the broader ‘technoscientific convergence that is taking place with biodigital technologies in the postdigital condition’ could come to transform how we live. This could bring us closer to ‘bioeconomic sustainability (Peters et al. 2021) and support how we discuss associated ‘ecopedagogies’ (Jandrić and Ford 2020). This is an evolutionary context where the NL community could surely undertake critical work in promoting connections through ecological learning designs that reflect this new context at its ‘point of intersection in educational praxis’ (Peters et al. 2021). Such contributions would extend the links that NL has established with critical pedagogy and ecologies of learning (Bozkurt, this paper) and ‘bring the importance of *learning to connect* to the fore [i]n order to develop more cohesive and sustainable societies’ (Carvalho, this paper). As Carvalho points out, ‘we need to understand that what happens at one side of the world, often affects and reverberates elsewhere, through a chain of interdependent links. We need to see ourselves as part of a global network.’ Directly connected to these changes, are questions like: ‘What new discourses and related behaviours might emerge through political bioeconomy? Rather than a dominant discourse about how technology will automatically enhance experience (as if experience were something universal that we all share), might we discuss new forms of ‘political bioeconomic discourse’? (Peters et al. 2021).

This is going to require a collective response where ‘partners involved in NL can be understood as *experimenting communities*’ who react even ‘while unfolding life and dealing with local and global challenges and fascinations’ (Thestrup and Gisley, this paper). If Covid-19 has taught us anything, it is that we cannot sit back and wait for the opportunity to contribute to an increasingly converged discourse. If NL is to be inclusive of all areas, such as ‘critical race studies, postcolonialism, indigenous knowledge, class, gender studies, queer theory, green and blue environmentalism and sustainability’ (Jones, this paper) and also rise to challenges relating to the Internet of Things, data, surveillance and algorithmic decision making, a collective response could prove more powerful than a fixed definition. Therefore, to reply as a dynamic, inclusive, ever-growing NL community, even as each new situation clarifies, is to keep developing critical responses in new empowering language. This means staying in motion with emerging postdigital-biodigital configurations (Peters et al. 2021) that are likely to provide a radically changed context for NL to contribute to, but which ‘intersect with NL’s focus to social justice and equality’ (Hadžijusufović, this paper) providing rich opportunities ahead.

## Review 1 (Laura Czerniewicz)

This community article initiated by the Networked Learning Editorial Collective is concerned with definitions (as the title makes explicit), with criteria for inclusion, and with principles. The most valuable part of the piece is the thread on principles. While language undoubtedly matters, it can also be appropriated to serve agendas at odds with their original meanings and intentions. One only has to consider how the concept of open education has been taken up by commercial publishers, promising access and inclusion in ways that are forms of openwashing (see Allen 2019; Jhangiani 2019). In the more extreme case of academic publishing, a prominent expert observed: ‘it is hard not to conclude that those of us who believed that open access … would lead to a fairer and more equitable scholarly communication system now look both naïve and silly’ (Poydner 2019).

Rather, then, it is the principles which are precious, not the name. And here the points made in the article are critical and encouraging. They articulate how to recognise what is—or could be—right in higher education in ways which are especially welcome at a time when so much is so wrong. The state of education post-Covid-19 is depressing when academics are losing their jobs, universities are closing, and suffering pervades the higher education sector everywhere. Also sobering is the growing critical scholarship unpicking, analysing, and exposing the ways that higher education is being financialised, learning experiences rendered as assets, universities reshaped by platform logic (see Komljenović 2020, 2021 and others). While this form of analysis is essential, it does not deal with aspirational principles.

What is refreshing about the article is that it sets out to reclaim and surface critical principles: that humanity is at the centre of educational technologies, that tools can be ‘convivial’ (Illich 1973), that knowledge forms should be inclusive. Community and connectedness are emphasized.

These qualities, call them criteria for being considered NL, however, need to be a means to an end rather than ends in themselves. An example makes the point. A community in South Africa described as having the attributes of liberty, voluntary co-operation, mutual respect, a tangible sense of community and shared purpose (Hogg 2015), sounds exemplary unless one is aware that they describe a whites-only town in the post-apartheid country (Webster 2019).

While implied, in order to strengthen the collective definition, it is necessary to articulate which goals these convivial tools, communities, and connections will serve. The public good. An alternative platform economy. Equity. Social justice. With these explicit goals and a bolder vision, the community definition will be a hopeful statement of what is, and can be, right, in digitally mediated Higher Education and the post-pandemic university.

## Review 2 (Jeremy Knox)

That this collective article is rather preoccupied with (re)defining NL is perhaps to be expected, given the ‘Inviting Redefinition’ (Networked Learning Editorial Collective 2020) article to which authors have been tasked with responding. However, the wealth of issues raised across these responses, rather than simply ‘enriching’ the NLEC definition, or indeed solving the question of ‘what is NL?’, combine in this article in a way that suggests something of an existential crisis for the term and its associated community. Across the paper, one finds petitions to redefine NL in terms of ‘affective dimensions’, ‘emergent, enacted, materialities’, ‘multimodal layer perspectives’, ‘power topologies’, and ‘spatial metaphors’. While this dizzying array of ideas is a tribute to the NL community’s ability to engage a diverse range of academic disciplines, it seems to work against the aim of the original paper: to express the ‘essence’ of NL in contemporary times.

The most insightful critiques of the NL definition come from Gourlay, who points out its ‘utopian nature’, and Lee and Bligh, who draw attention to ‘the dynamics of struggle in daily practice’. Both these sections identify what is perhaps the most obvious blind spot in the NL definition(s): the assumption that all the connection, collaboration, and interaction with technology is, and should be, *congenial*, even *pleasurable*. In contrast, the agonism implied by Lee and Bligh appears to serve as a much better definition for understanding the everyday ‘lived experiences’ in which NL might take place.

However, ultimately, this collection of responses could have demonstrated more critical reflection on the very practice of definition itself. It is certainly admirable that across these responses there is a collective attempt to include so many important contemporary concerns; however, it ends up rendering the practice of defining NL something of a Sisyphean task, each time endeavouring to arrive at the top of the hill with a perfect, all-encompassing definition. Where Tickner suggests ‘universal design, cultural inclusion, and digital equity’ and Thibaut, ‘the inclusion of global, local and sustainable views’, one wonders how accommodating a definition can be before it starts to lose its grip on the specificities of the very real and divergent contexts in which NL is necessarily situated. Conversely, as Bayne’s contribution emphasises, the problems do not go away when one tries to tighten the definition: ‘[t]o define a field is necessarily to put boundaries around it’.

Bayne’s ‘trap’ of endlessly defining NL might be avoided by putting NL ‘to work’, rather than trying purify it; doing something with it, rather than struggling to draw its boundary. Here the NL community might look to other areas of theory that have attempted to move beyond the impasse of ideology. To borrow a phrase from Deleuze and Guattari (1987), how might we ‘plug in’ NL to other concepts, such as postcolonialism? To reuse a term from Haraway (1997), how might we ‘diffract’ NL through social justice theory? In other words, to allow the *concept of NL itself* to become ‘networked’: to make connections, to interrelate, to transform, mutate, and hybridise in response to the pressing issues of our time.

## Footnotes

1 See https://www.networkedlearning.aau.dk/nlc2020/. Accessed 28 January 2021.

2 See https://www.springer.com/series/11810. Accessed 28 January 2021.

3 For a genealogy of the definition, as well its variants, see Networked Learning Editorial Collective (2020).

4 This model identifies a person’s health strengths and challenges in relation to four essential interrelated aspects, Taha Whānau (family and relational), Taha Tinana (physical), Taha Hinengaro (emotional) and Taha Wairua (spiritual wellbeing).
